# The Cell Theories of Huxley and Virchow

**Published:** 1870-10

**Authors:** I. N. Danforth

**Affiliations:** Chicago


A MONTHLY RECORD OF

*Medicine, Surgery, and the Collateral Sciences.*

EDITED BY J. ADAMS ALLEN, M.D., LL.D.; AND WALTER HAY, M.D.

VOL. XXVII.—OCTOBER, 1870.—No. 10.

### Original Communications.

ARTICLE I.—*The Cell Theories of Huxley and Virchow.* By I. N. DANFORTH, M.D., Chicago.

The student who reads the writings of Prof. T. H. Huxley will at once recognize the fact that he is in the presence of a *master* of terse and vigorous composition, as well as of clear, concise and cogent reasoning; but he will also find himself confronted by the stern necessity of turning his back upon this remarkable author, or else of bidding farewell to all our notions of bygone years regarding vital force. Mr. Huxley does not believe in vital force, as something superadded to, and working through, matter. He is, moreover, regarded as the "ablest English advocate" of Darwinism, particularly "with reference to its application to the human race, which he believes to be nearly related to the higher apes." His more recent views regarding "Protoplasm, or the Physical Basis of Life," are well known to the medical world, and his doctrines regarding the cell are quite consistent with the "physical" tendency of his faith and philosophy.

Mr. Huxley's first article on the cell, so far as I am aware, was published in the British and Foreign Medico-Chirurgical Review for October, 1853. It was very widely read in England, and also to a very considerable extent in this country, but I think its conclusions found little favor on either side of the Atlantic, except that in England they were loudly trumpeted by the "advanced" school of philosophers whereof Mr. Huxley himself has now come to be the "Head Centre."

In this article, Mr. Huxley adopts to a considerable extent, and expounds at length, the doctrines of C. F. Wolff, first announced in the year 1759; thus rescuing this well-nigh forgotten author from the shades of impending oblivion.

Notwithstanding the intrinsic errors contained in Wolff's theory, it answered well as a sort of stepping-stone to the more recent notions of Huxley, and it is easy to trace the gradual growth of the former into the latter. To Wolff, the cell is a *passive*, not an *active* body; it is a mere cavity formed in and by the "blastema" or surrounding fluid; it possesses no organizing or developmental power, but is itself the visible result of the action of the organizing power inherent in the living mass, or what Wolff calls the *vis essentialis*. For him, however, this *vis essentialis* is no *archaeus*, but simply a convenient name for two facts which he takes much trouble to demonstrate: "the first, the existence in living tissues (before any passages are developed in them), of currents of the nutritious fluid determined to particular parts, by some power which is independent of all external influence; and the second, the peculiar changes of form and composition, which take place in the same manner." (Huxley.) Thus far we have the views of Wolff, as expounded and adopted by Mr. Huxley; and thus far we find the cell shorn of all essential power, and reduced to a mere passive body, which owes its existence to the *vis essentialis* resident in the blastema or fluid in which it floats, and of necessity holding its tenure of existence subject to the option of the same power; furthermore this *vis essentialis* is "simply a convenient name" for describing the visible phenomena which take place during the process of organization.

From this basis, Prof. Huxley goes on to complete his theory: *First*, "there is a condition of all kinds of living matter, in which it is an *amorphous germ*—that is, in which its external form depends merely on physical laws, and in which it possesses no internal structure,"—and, "according to the nature of certain previous conditions," will be the future changes or differentiations which this "amorphous germ" shall undergo.

*Secondly*, the "morphological differentiation may be of two kinds." "In the lowest plants and animals," it is external—that is, concerning the outward shape of the germ, and having nothing to do with internal structure.

*Thirdly*, in all other plants and animals the germ "*becomes separated* into a certain central portion, which we have called the *endoplast*, and a peripheral portion, the *periplast*"—and the "germ in this state constitutes a vesicle with a central particle, or a nucleated cell." The molecular forces, or "*vis essentialis*," are not localized either in endoplast or periplast; there is no attraction or influence exercised by one over the other; the changes which each subsequently undergo, have no connection with each other, but each proceeds "in accordance with the general determining laws of the organism."

*Fourthly*, "The endoplast grows and divides," but generally "undergoes no other morphological change." "So far from being the centre of activity of the vital actions, it would appear much rather to be the *less important histological element*."

*Fifthly*, the *periplast* (cell-wall, contents, and intercellular substance) "is the subject of all the most important metamorphic processes, whether morphological or chemical, in the animal and plant. By its differentiation, every variety of tissue is produced; and this differentiation is the result, not of any metabolic action of the endoplast," but of molecular changes in its substance under the guiding force of the "*vis essentialis*."

*Sixthly*, the metamorphoses of the periplastic substance are two-fold—*chemical* and *structural*. The former relate to *conversion*, as of cellulose into xlyogen; or to *deposit*, as of silica in plants, and calcareous salts in animals. The structural metamorphoses are also two: *vacuolation* or the formation of cavities, as in the intercellular passages of plants, the first vascular canals in animals, and *fibrillation*.

So much for Mr. Huxley's doctrines in 1853. It is noticeable that, at this time, he admitted the existence of a guiding or organizing force—"the *vis essentialis* of Wolff, or the vital forces of the moderns," which, whatever he may have regarded its nature, was certainly to him something superior to and superadded to the ordinary forces resident in matter. It will be seen that in this regard he has essentially modified his views since the date above-mentioned. In one particular, however, as Dr. Tyson has pointed out, Mr. Huxley has been misunderstood by some of his critics, especially Prof. Beale, namely, in that he has been represented as saying that the *periplast* is of itself active. If I correctly understand Mr. Huxley, he means that the *periplast* is *acted upon* by the *vis essentialis;* that it is the subject of action, rather than itself the active agent, and, in the light of Prof. Huxley's recent teachings, it is important that this matter be correctly understood.

It is scarcely necessary to enter into a lengthy explanation of the later views of Mr. Huxley, since they have been very generally read by the profession, and are readily accessible to everybody. Briefly, they are as follows: There is an albuminous matter, common to every form of life, whether plant or animal; this matter is "Protoplasm, or the Physical Basis of Life;" it "presents a three-fold unity—namely, a unity of power or faculty, a unity of form, and a unity of substantial composition," wherever and whenever found; this protoplasm or matter of life exists in the form of nucleated cells, variously modified as to form and size to fulfill various functions; plants can "manufacture" fresh protoplasm from its chemical elements, without limit, but animals must obtain it ready made, and hence, are dependent "in the long run" upon the vegetable world; the protoplasm of one animal may readily be converted or "transubstantiated" into that of any other animal, and this "fact speaks volumes for the general identity of that substance in all living beings."

But Mr. Huxley's views concerning vital action have been most widely and sharply criticised. According to him, there is no mysterious, essential power superadded to the ordinary chemical and molecular forces inherent in protoplasm, which we can call vitality. The phenomena exhibited by it are simply its properties, precisely as the phenomena of water are *its* properties, and we may as philosophically and as truthfully account for the properties of water by ascribing them to a mysterious power and call it "aquosity," as to account for the properties of protoplasm by ascribing them to a like mysterious power and call it "vitality." "What better philosophical status has 'vitality' than 'aquosity'?" asks Mr. Huxley—a question, by the way, which it is not difficult to answer. These singular notions were announced in a public lecture in Edinburgh, in November, 1868. Although they have achieved a very wide circulation, they have found few adherents among scientific men, with the exception of the school of so-called "physicists," few in number, by whom they were of course joyfully welcomed. Nor is it probable that time and future investigations will render them more acceptable to the great body of scientists throughout the world.

Among modern observers and writers, no one has exerted a wider influence in the domain of cytology than Prof. Virchow, the eminent pathologist of Berlin. As I have already remarked, his views were, to a considerable extent, anticipated by that most excellent observer, Prof. John Goodsir; although it remained for Prof. Virchow to complete Goodsir's investigations, and place the results thereof in a systematic and accessible form; a work which is very fully and ably accomplished in his "Cellular Pathology," published in 1858.

The characteristic views of Virchow in regard to cell growth and cell action, seem to be but little known in this country, notwithstanding the fact that his book has met with a very extensive sale. I shall therefore attempt to state them briefly.

*First*, The cell is the only possible starting point for all vital processes, whether morbid or healthy; it is never developed, except from a pre-existing cell—or primarily from the ovum; hence *omne vivum ex ovo* becomes, in its broader signification, *omnis* cellula e cellulâ, and this is equally true of plants and animals; the cell consists essentially of "cell wall," "cell contents," and "nucleus"—the nucleolus not being considered essential. The nucleus is exclusively concerned in the *life* of the cell; upon it depends the maintenance of the present, and development of the future cell; while the cell contents (protoplasm of Huxley; formed material of Beale) are entirely concerned in *function*, as contraction, secretion, etc. With regard to "free cell development," it has, says Virchow, "been almost entirely abandoned, and in support of the correctness of which, not one fact can, with certainty, be adduced." (Cellular Pathology, page 36.) According to this view, the cell is the seat of all physiological and pathological processes, rather than the blood, or the nervous centres; hence the term "cellular pathology," in preference to humoral, neural or solidistic pathology.

*Secondly*, "Every animal presents itself as a sum of vital unities, every one of which manifests all the characteristics of life." * * * Hence there exists in the body "a kind of social arrangement of parts," wherein a number of "individual existences are mutually dependent, but in such a way that every element (cell) has its own special action. and, even though it derive its stimulus to activity from other parts, yet alone effects the actual performance of its duties." In following out this conception, Virchow "portions the body out into cell territories," or innumerable minute islets, which are conceivably tributary to, or are "ruled over" by a cell or cells adjacent. This is more particularly marked in some pathological new formations, consisting largely of intercellular substance, in which the boundaries of the cell districts are very "sharply defined."

*Thirdly*, The connective tissue corpuscle is not a nucleus alone, as originally stated by Schwann and others, but a complete cell (cell-wall, contents and nucleus); this cell is the starting point of all healthy and morbid growth, except such as may be attributed to epithelial origin; from the "proliferation of this cell, result pus, cancer, tubercle, the various tumors, and, indeed, all pathological new formations, with the exception above-mentioned. Hence these products are not exudations from the blood, but are of local origin—the result of increased or perverted cell-action, expressed by the word "proliferation."

*Fourthly*, The last and most doubtful feature of Virchow's theory relates to his peculiar system of canals or tubes, resulting from the anastomosis of cells. This is particularly the case with connective tissue cells, concerning which Virchow says, "It may with some degree of certainty be demonstrated, that these anastomoses constitute a peculiar system of tubes or canals which must be classed with the great canalicular system of the body, and which particularly, forming as they do, a supplement to the blood and lymphatic vessels, must be regarded as a new acquisition to our knowledge, and in some sort filling up the vacancy left by the old 'vasa serosa' which do not exist." This reticular arrangement is said to be "possible in cartilage, connective tissue, bone and mucous tissue in the most different parts." (Cellular Pathology, page 76.)

This system of tubuli is also believed to extend to and include the tortuous fibres of the yellow elastic tissue, and the dentinal tubules. But the majority of observers have thus far failed to discover these anastomosing cells, and the resulting tubules, and the doctrine has not been received with general favor, even in Germany. It would seem that a point so important might be easily, speedily and conclusively settled; but it is extremely difficult, in microscopical investigations, to eliminate the various sources of optical delusion, and decide really what is true and what is false; and in this way, probably, either Virchow or his opponents have been grossly deceived.

Prof. L. S. Beale is a decided disbeliever in the existence of these tubules, and his statements are always entitled to great credence. But, considered in their entirety, the teachings of Virchow are vastly important in their relations to pathology. Whatever their ultimate value may prove to be as regards the establishment of new facts, and the enlargement of the bounds of our knowledge, this much is certain: the labors of Virchow have established a new order of things in regard to care and painstaking accuracy in prosecuting minute investigations; conclusions are less hastily jumped at; crude and silly theories are less frequently spawned upon the world; and the whole tendency of histological inquiry has changed for the better, in that more of care, more of accuracy and more of close and thoughtful study have been infused into the labors of observers everywhere, since the methodical writings of Virchow have become the common property of the profession.

